# OP51 Strategies For Training Laypeople To Participate In Health Technology Assessment: A Scoping Review

**DOI:** 10.1017/S0266462324001132

**Published:** 2025-01-07

**Authors:** Quenia Cristina Dias Morais, Alex Itaborahy, Leny Frossard, Iandy Mateus, Bianca Leite, Marisa Santos

## Abstract

**Introduction:**

This study aimed to map strategies for educating laypeople about health technology assessment (HTA). Although integrating community is challenging, the engagement of patients/public in the processes of HTA has garnered support and endorsement from international network agencies. Dissemination of information, educational empowerment, and training are vital to give individuals capacity to partake in the intricate web of processes actively.

**Methods:**

This review considered studies addressing educative strategies to train laypeople on HTA, additionally mapping and summarizing relevant methodological papers from any international HTA agency. Four databases were searched for qualitative, quantitative, and mixed methods study designs. The grey literature search included policy and practice documents from HTA and health organization websites. Two reviewers independently completed title and abstract screening before the full-text review and data extraction.

**Results:**

The main contributors to the production of knowledge about educating laypeople in HTA were the United Kingdom (40%), Spain (20%), and Canada (13%). Most studies included were conducted in the context of the United Kingdom (27%), followed by Spain (20%), and international networks context (20%). The main strategies included conference-like events (21%), the production of educational materials (18%), training (11%), and the use of plain language (8%). Furthermore, international HTA and health agencies have offered courses, and online training produced and made available online guidance materials for increasing laypeople’s participation in the HTA process.

**Conclusions:**

Despite the global efforts to educate laypeople on HTA, jurisdictional variations underscore the need for a more inclusive approach. Strategies like events, educational material production, training, and clear-language use offer diverse avenues for public engagement. International agencies’ commitment to courses, online training, and guidance reflects a collective effort to enhance public involvement.

